# Adipose tissue macrophage in obesity-associated metabolic diseases

**DOI:** 10.3389/fimmu.2022.977485

**Published:** 2022-09-02

**Authors:** Jingfei Yao, Dongmei Wu, Yifu Qiu

**Affiliations:** ^1^ Institute of Molecular Medicine, Beijing Key Laboratory of Cardiometabolic Molecular Medicine, College of Future Technology, Peking University, Beijing, China; ^2^ Peking-Tsinghua Center for Life Sciences, Peking University, Beijing, China

**Keywords:** adipose tissue macrophage, obesity, adaptive thermogenesis, insulin resistance, fibrosis, adipokines

## Abstract

Adipose tissue macrophage (ATM) has been appreciated for its critical contribution to obesity-associated metabolic diseases in recent years. Here, we discuss the regulation of ATM on both metabolic homeostatsis and dysfunction. In particular, the macrophage polarization and recruitment as well as the crosstalk between ATM and adipocyte in thermogenesis, obesity, insulin resistance and adipose tissue fibrosis have been reviewed. A better understanding of how ATM regulates adipose tissue remodeling may provide novel therapeutic strategies against obesity and associated metabolic diseases.

## Introduction

Obesity is an accumulation of adipose tissue resulting from an energy imbalance, which has been linked to numerous comorbid conditions including type 2 diabetes mellitus, nonalcoholic fatty liver disease (NAFLD), atherosclerosis, cancers as well as COVID-19 (1, 2). Adipose tissues, which include brown adipose tissue (BAT) and white adipose tissue (WAT), play critical roles in the maintenance of energy homeostasis. WAT store energy when nutrition is abundant, while BAT dissipate energy for heat production through a mitochondrial uncoupled respiration.

Besides adipocytes, many types of immune cells reside in both BAT and WAT to control adipose tissue homeostasis (3). Among these immune cells, macrophage is the most abundant population, constituting 5%-10% cell numbers of the adipose tissue in the lean state and increasing to 50% or more in the condition of extreme obesity both in humans and in mice (4). Macrophage is derived from embryo or adult bone marrow-derived circulating monocytes, which are essential in the maintenance of tissue homeostasis and play a vital role in different pathologies. Macrophage is a heterogeneous population of immune cells, such as Kupffer cell in liver, alveolar macrophage in lung, microglia in brain among many others. They play tissue-specific functions in homeostatic and immune-related responses shaped by different local microenvironment (5, 6).

Adipose tissue is an energy reservoir which contains lots of lipids and acts as an important endocrine organ by secreting numerous factors. These lipids and factors generate a specific microenvironment that distinguishes adipose tissue from others and distinguishes adipose tissue macrophage (ATM) from macrophage in other tissues. There are two types of activated macrophage in adipose tissue, named M1 macrophage and M2 macrophage. In the adipose tissue of lean mice, most macrophages are M2 activated, which produce anti-inflammatory cytokines including interleukin-10 (IL-10) and TGF-β, contributing to resolution of inflammation and tissue homeostasis. But in obese mice, the adipose tissue recruits many M1 macrophages, which generate proinflammatory cytokines, causing adipose tissue inflammation and metabolic dysfunction (7, 8).

Here, we summarize the latest progresses of the metabolic implications of ATMs. We describe the polarization and recruitment of adipose tissue macrophages, and discuss their functions both in health and metabolic diseases, including thermogenesis, obesity, insulin resistance as well as adipose tissue fibrosis.

## Macrophage polarization

**Figure 1:**
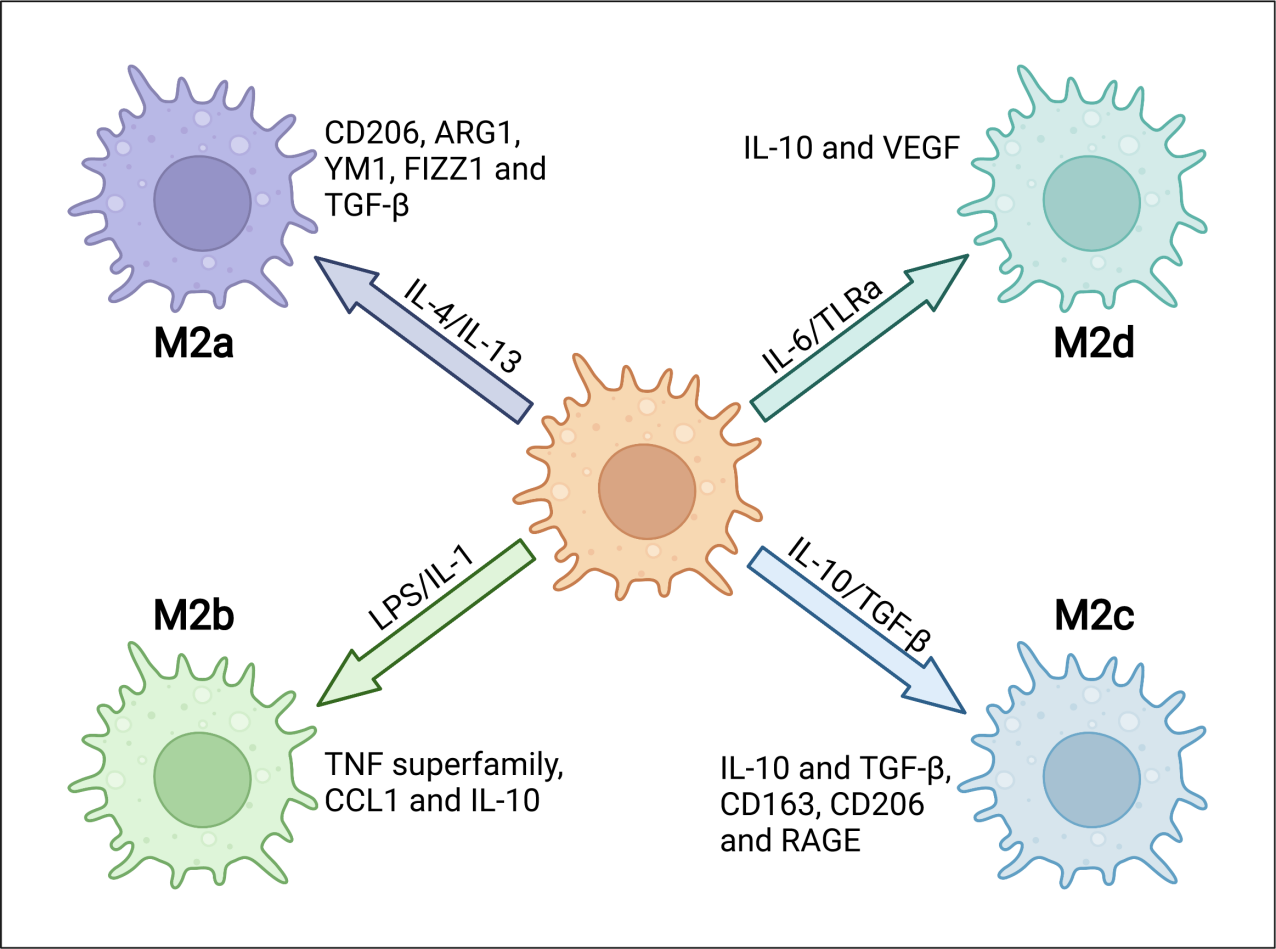
The heterogeneity and characterizations of M2 macrophages. M2 macrophages can be subgrouped into M2a, M2b, M2c and M2d depending on different microenvironmental stimuli. Specific stimuli include, but are not limited to, IL-4 or IL-13 for M2a, LPS or IL-1 receptor ligands for M2b, IL-10 or TGF-β for M2c and IL-6 or Toll-like receptor agonists (TLRa) for M2d. Different subtypes express distinct markers, including intracellular proteins and secreted cytokines.

M1/M2 polarization of macrophage is a process by which macrophages produce distinct functional phenotypes driven by microenvironmental stimuli in specific conditions. There are two types of macrophage polarization in adipose tissue, M1 and M2 macrophages. M1 macrophages are generated when stimulated with lipopolysaccharide (LPS) or Th1 proinflammatory cytokines such as IFN-γ. Meanwhile, M2 macrophages are induced by Th2 cytokines such as IL-4 and IL-13. M1 macrophages are usually characterized by enhanced phagocytic activity and increased secretion of proinflammatory cytokines (9). Phenotypically, M1 macrophages show enhanced expression of main histocompatibility complex class II (MHC-II), CD68, CD80 and CD86 both in mice and humans (10). These characteristics are mainly promoted by IFN-γ-mediated Janus kinase-signal transducer and activator of transcription (JAK-STAT) signaling or directly by pathogen associated molecular patterns (PAMPs) such as LPS. Thus, M1 macrophages, along with other innate immune cells, provide the first line of defense to fight against infectious pathogens and promotes Th1 immune response. Several pathways have been discoveried to regulate M1 activation. Transcription factor interferon regulatory factor 5 (IRF5) has been reported as a key player in the polarization of both human and mouse macrophages towards a proinflammatory M1-like phenotype by controlling expression of M1 markers, as well as Th1 and Th17 responses (11). STAT1, which is activated by LPS/TLR4 pathway, plays a critical role in M1 polarization (12, 13). Suppressor of cytokine signaling 3 (SOCS3) activates nuclear factor kappa-light-chain-enhancer of activated B cells (NF-κB) pathway to produce NO, which promotes expression of M1 markers and inhibits IL-10 expression (14). M2 macrophages have been initially identified during helminth infection, which promotes a Th2-polarized response. They are usually characterized by the expression of M2 markers including arginase 1 (ARG1), chitinase 3-like 3 (also known as YM1), FIZZ1 and CD206. Depending on the contexts and the expression of phenotypic markers, M2 macrophages can be subtyped into M2a, M2b, M2c and M2d ones (15, 16). M2a macrophages play a role in the Th2 response during parasite infections. They are typically induced by stimulation of IL-4 and IL-13, which are produced by eosinophils. M2a macrophages are characterized by high surface expression of CD206, ARG1, YM1, FIZZ1 and TGF-β, and they can promote fibrosis and wound healing. M2b macrophages show immune-regulated and anti-inflammatory effects which induced by IL-1 and TLR agonists such as LPS, expressing high levels of TNF superfamily, C-C motif chemokine ligand 1 (CCL1) and IL-10.M2c macrophages are induced in the presence of IL-10, TGF-β and glucocorticoids. They are usually considered as deactivated or anti-inflammatory macrophages, and involved in phagocytosis of apoptotic cells. M2c macrophages secret large amounts of IL-10 and TGF-β, and express multiple markers including CD163, CD206, RAGE and other scavenger receptors. M2d macrophages, also known as tumor-associated macrophages (TAMs), are induced by the TLR antagonists, and they release IL-10, TGF-βand vascular endothelial growth factors (VEGF) to contribute to tumor angiogenesis (17–21) (**Figure 1**). Transcription factors such as Krueppel-like factor 4 (KLF4), STAT6 and peroxisome proliferator-activated receptor-γ (PPARγ) are all involved in the polarization of M2 macrophages (22–24). Besides, recent studies identified PI3K/AKT signaling as another critical mediator in mouse M2 macrophage polarization, which is independent of the well-established JAK1/STAT6 pathway (25). IL-4 stimulates the phosphorylation of IRS-2 that leads to the recruitment and activation of PI3K/AKT pathway (26). Interestingly, different AKT isoforms seem to play different roles in macrophage polarization, with Akt1 isoform deficiency leading to an M1 activation while Akt2 isoform ablation causing an M2 phenotype (27).

In obese adipose tissue, ATMs tend to polarize to M1 macrophages, which are mainly regulated by adipocytes. Adipocytes exert effects on ATM phenotypes *via* a variety of mechanisms. Obese adipocytes secrete many proinflammatory cytokines, including monocyte chemoattractant protein 1 (MCP-1/CCL2), which recruit macrophages and induce their polarization to proinflammatory M1 type. Besides cytokines, ATM polarization is affected by lipids and glucose which are much more abundant in the obese condition. Macrophages treated by very low-density lipoproteins (VLDLs) and short chain fatty acids increase a secretion of proinflammatory cytokines (28). Free fatty acids (FFAs), which is increased in the serum of obese animals, can induce TLR4 signaling activation in murine ATMs and polarize ATM to M1 (29–31). High levels of glucose directly promotes macrophage M1 activation *via* the Rho-associated protein kinase (ROCK)/c-Jun amino-terminal kinase (JNK) and ROCK/extracellular signal-regulated kinase (ERK) pathways (32, 33). Besides, miR-155, which is secreted by adipocyte-derived microvesicles (ADM), regulates M1 macrophage polarization (34). Moreover, DPP4, a dipeptidyl protease expressed and released by hapatocytes, can activate ERK1/2 and NF-κB signaling to induce MCP-1 and IL-6 expression in ATMs that promotes adipose tissue inflammation (35).

Lean adipocytes normally secrete adiponectin that stimulates M2 ATM polarization (36). PPAR-δ, a nuclear hormone receptor, plays an important role in the activation of M2 macrophage, and alleviates diet-induced insulin resistance (37). PPAR-δ is induced by cellular lipids when apoptotic cells are engulfed by macrophages and further regulates the clearance of these apoptotic cells (38). Helminth infection significantly promotes Th2 responses and M2 macrophage polarization, which alleviate obesity. Adoptive transfer of M2 macrophages treated by helminth to recipient mice significantly improve high-fat diet (HFD)-induced obesity (39). Furthermore, PPAR-γ has been reported to polarize human monocytes to M2 macrophages *in vitro* (40), while deletion of PPAR-γ in myeloid cells inhibits M2 macrophage activation and accelerates diet-induced obesity and insulin resistance in mice (41).

A number of regulators govern the polarization of M1 or M2 macrophage and the switch between M1 and M2 activation. TLR4 is the key component in LPS-mediated M1 polarization, and TLR4 deficiency inhibits HFD-induced recruitment of proinflammatory M1 macrophages and induces M2 macrophage polarization (42). 11β-HSD1, a reductase reactivating glucocorticoids, was reported to promote the switch from M2 to M1 macrophages in human obesity (43). In addition, inositol-requiring enzyme 1α (IRE1α) wasalso reported to be a key factor controlling ATM polarization and energy balance in mice. Deficiency of IRE1α promotes M2 macrophage polarization, and transcriptomic profiling revealed that expression of IRF4 and KLF4 can be inhibited by IRE1α, both of which are critical players controlling M2 polarization (44). MicroRNA has rencently been implicated in the regulation of macrophage M1/M2 polarization as well as insulin resistance. miR-495 promotes M1 macrophage activation by targeting and inhibiting the expression of *Fto* (45). However, another study reported that *Fto* silencing significantly suppressed both M1 and M2 polarization, through inhibiting the expression of STAT1 and of STAT6 and PPAR-γ respectively (46). *FTO* gene has been considered as the strongest genetic effector in human polygenic obesity, in which IRX3 may participate by mediating this effect. Recently, using cell-specific knockout mouse models, we demonstrated that macrophage IRX3 regulates body weight through acting as a transcriptional factor to control the expression of proinflammatory cytokines (47). Mechanistically, we found that IRX3 promotes M1 but not M2 gene expression when it is phosphorylated and activated by JNK1/2 in macrophages (47).

## ATM recruitment

Healthy adipose tissue predominantly contains anti-inflammatory M2 macrophages that originate from yolk sac, with a little contribution of circulating monocytes (48). An extreme increase in adipocyte size is accompanied by an inadequate supply of oxygen due to expanding adipose tissue, causing an increased frequency of adipocyte death and following macrophage recruitment (49, 50). Over 90% of macrophages recruited to adipose tissue are arranged around dead adipocytes, which form a structure called “crown-like structure (CLS)”, both in obese animals and humans (49, 51). These recruited macrophages exert their phagocytotic function to clear dead adipocytes. Deficiency of mannose-binding lectin (MBL), which can bind apoptotic cells and promote engulfment by phagocytes, inhibits the clearance of apoptotic cells in adipose tissue (52). Meanwhile, macrophages in CLS store and buffer excess lipids released from dead adipocytes, which are named lipid-laden macrophages (53). The number of CLS is highly positively correlated with adipose tissue inflammation and metabolic disorders of obese subjects (54, 55). Proinflammatory adipokines including MCP-1 and TNF, as well as saturated fatty acids secreted by obese adipocytes, can recruit and activate ATMs (56). Activated macrophages release proinflammatory chemokines including MCP-1 to recruit more monocytes from blood into adipose tissues by binding to its receptor C-C chemokine receptor type 2 (CCR2) (37). After infiltrating into the adipose tissue, monocytes differentiate to macrophages and interact with adipocytes in a paracrine manner, further increasing the secretion of proinflammatory cytokines (57). This interaction between adipocytes and macrophages establishes a vicious spiral in obese adipose tissue and persistently recruits more and more macrophages from circulation (55). Besides, obesity promotes the expression of chemokine receptors in adipose tissues from both mice and humans, which further enhance the vicious spiral (58, 59).

Many cytokines and their receptors participate in the recruitment of monocytes/macrophages. As previously mentioned, MCP-1-CCR2 is reported as the most important cascade in macrophage recruitment. MCP-1 in adipocytes promotes ATM recruitment and insulin resistance in mice (57), while HFD-induced macrophage accumulation in adipose tissue was extensively reduced in MCP-1 KO mice (60), which indicates a critical role of MCP-1 in the trafficking of macrophages. In addition to adipose tissue, the MCP-1-CCR2 circuit plays an important role in recruiting monocyte in many other tissues, such as in liver, heart and lung (61–64). Moreover, MCP-1 has been reported to induce local proliferation of macrophages, which is another important mechanism underlying obesity-elicited macrophage accumulation (65). Besides MCP-1, CCR2 can also be activated by other ligands, including CCL7 (MCP-2) (66), CCL8 (MCP-3) (67), CCL13 (MCP-4) (68), and CCL12 (MCP-5) (69), many of which are expressed in obese adipose tissue and affect monocyte/macrophage recruitment (58). On the other hand, CCR5 expression is highly upregulated in obesity and FACS analysis further illustrated that WAT from obese mice have significant accumulation of CCR5 positive macrophages. Consistently, CCR5 deficient improves obesity-induced insulin resistance in mice (70). CCL3 and CCL5 have been reported as ligands of CCR5 (71). Inhibition of CCL3 reduces macrophage infiltration and activation by downregulating CCR5 (72). CCL5 recruits macrophages mainly by promoting cell adhesion and transmigration of monocyte vascular endothelial cells (73). CX3CL1-CX3CR1 axis also precipitates in macrophage infiltration and inflammation in both atherosclerosis and rheumatologic disorders (74, 75). It has been suggested that adipocytes express CX3CL1 that can activate the CX3CR1 signaling in macrophages (76).*Cx3cr1*-deficient mice fed HFD displayed significantly declined monocytes and produced less proinflammatory cytokines in the WAT (77). However, another study reported that *Cx3cr1*-dificient mice showed a reduction of M2-polarized macrophage migration, and exacerbated adipose tissue inflammation, insulin resistance and hepatic steatosis when fed HFD (78). Serum amyloid A (SAA) promotes monocyte recruitment by inducing the expression of the adhesion antigens CD11b, intracellular adhesion molecule-1 (ICAM-1) and vascular adhesion molecule-1(VCAM-1) through a NF-κB-dependent signaling (79, 80). Myeloid cell-specific ablation of GPR105, which is activated by UDP and UDP-linked glucose, prevents macrophage recruitment to liver or adipose tissue in mice fed HFD (81). C-X-C motif chemokine ligand 14 (CXCL14), which is required for the activation of dendritic cells, is another chemoattractant participates in the recruitment of macrophages into adipose tissue and insulin resistance, although its receptor has not yet been identified (82, 83). Using knockout mouse model, CXCL14 has recently been reported to be produced by brown adipocytes upon thermogenic activation and promotes the recruitment and activation of M2 macrophages in BAT (84).

**Figure 2:**
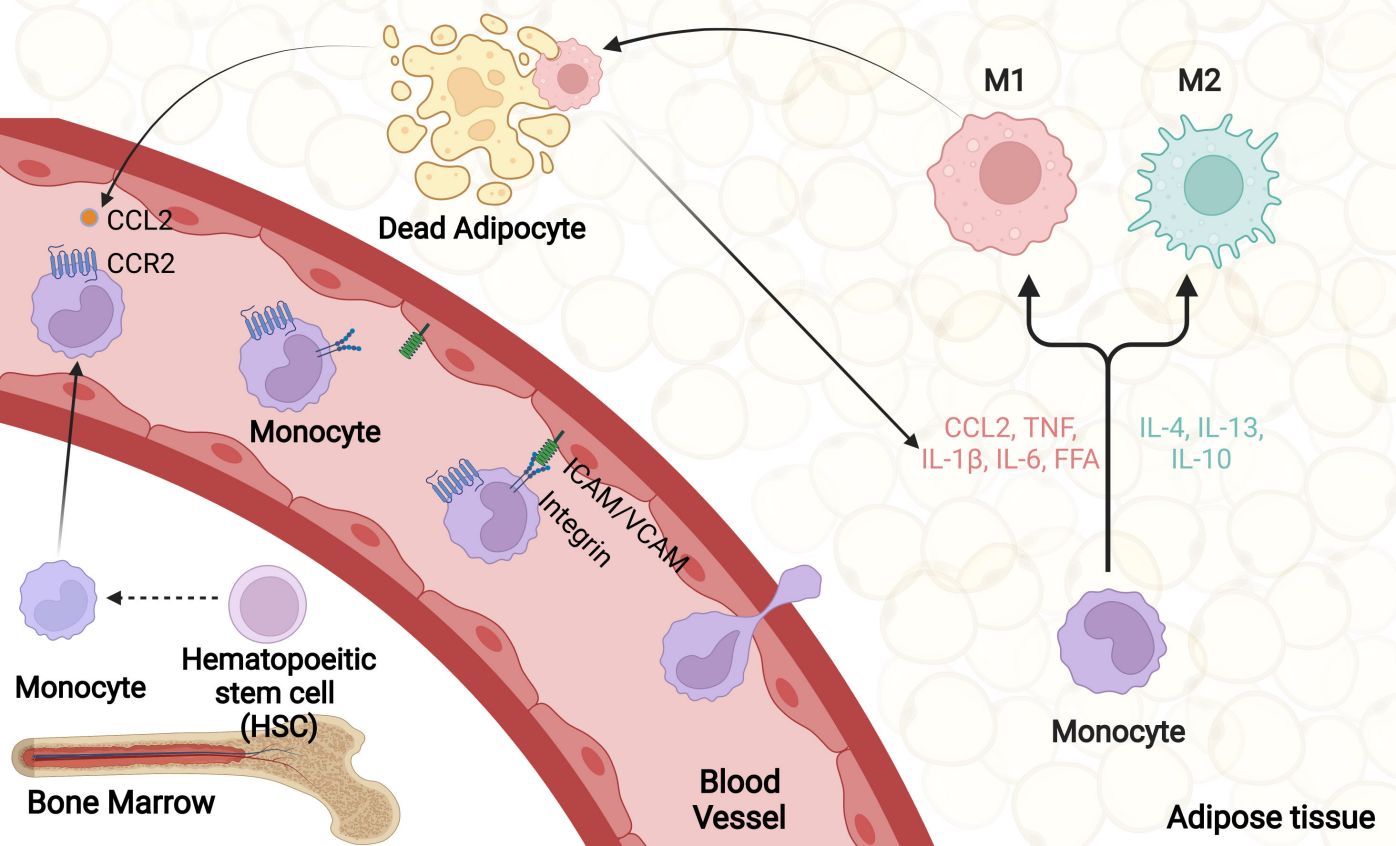
ATM recruitment. Obesity promotes adipocyte death as well as adipose tissue inflammation, which firstly trigger a large number of chemokines secretion, including CCL2. Upon activation, monocytes and vascular endothelial cells produce various cellular adhesion molecules, mainly integrin, ICAM-1 and VCAM-1. Through rolling and adhesion process, monocytes bind to adhesion molecules on vascular endothelial cells and transmigrate from blood vessel to target adipose tissue. Eventually, recruited monocytes differentiate into proinflammatory macrophages in response to local microenvironmental stimuli.

Collectively, there are several steps in the recruitment of ATMs. Initially, Obesity-induced adipocyte death and adipose tissue inflammation promote a secretion of CCL2 and other chemokines, which bind to their receptors on monocytes circulating in the blood. Then, activated monocytes adhere to endothelial cells of blood vessel *via* upregulated adhesion molecules including ICAM-1, VCAM-1 and integrin. After integrin-dependent lateral migration, monocytes transmigrate from blood vessel to target adipose tissue. Eventually, recruited monocytes differentiate into proinflammatory macrophages in response to local microenvironmental stimuli (**Figure 2**) (85).

## ATM in adaptive thermogenesis and lipolysis

Brown adipocyte and beige adipocyte, which highly express mitochondrial uncoupling protein 1 (UCP1), are responsible for adaptive thermogenesis and protect against metabolic diseases in mice and humans. FFAs, which are produced as a result of lipolysis, serve both as direct activators of UCP1 and fuel sources for thermogenesis (86). Thermogenesis and lipolysis of adipose tissues and browning of WAT are dynamic processes, in which both M1 and M2 macrophages play critical roles.

### M2 macrophages in adaptive thermogenesis and lipolysis

M2 macrophage has been demonstrated as an activator to promote fat thermogenesis and lipolysis through different mechanisms. In 2011, cold-induced BAT thermogenesis and WAT lipolysis were first linked to macrophage M2 recruitment and activation (87). Using myeloid cell-specific ILR4α-deficient and IL4 administration mouse models, M2 macrophage has been revealed to be required and sufficient for BAT and beige fat thermogenesis and lipolysis (87, 88). Mechanistically, cold exposure induces the expression of tyrosine hydroxylase (TH) and resultant catecholamine production in M2 macrophages to sustain thermogenesis and lipolysis, although whether M2 macrophages expresse a significant amount of TH is under debate (87–90). Another study reported a similar recruitment and pro-thermogenic effects of M2 macrophage in cold-induced browning of subcutaneous white adipose tissue (scWAT) in mice, further supporting a critical role of M2 macrophage in adaptive thermogenesis (88, 90). Moreover, CD44^+^ M2 macrophage is recruited by CL316.243 (CL)-mediated adipocyte death to produce high level of 9-hydroxyoctadecadienoic acid (9-HODE) and 13-HODE, two known PPARγ ligands, which promote differentiation of platelet-derived growth factor receptor alpha (PDGFRα^+^) progenitors to beige adipocytes (91). Besides, macrophage-derived osteopontin (OPN) triggers a recruitment of PDGFRα^+^ progenitors, which contribute to beige adipogenesis (92). In addition to the above paracrine manner, M2 macrophage has also been reported to promote beige adipogenesis in a direct-contact manner both in humans and mice (93). More recently, it has been reported that brown adipocyte ejectes damaged mitochondria *via* extracellular vesicles, whose removal by M2 macrophage ensures optimal BAT thermogenesis in mice (94). Started from the M2 macrophage, multiple studies have explored the importance of other anti-inflammatory cytokines in the regulation of adipose thermogenesis and lipolysis.IL-4, IL-13 and IL-33, as key members in type 2 cytokines, all have been demonstrated to promote thermogenesis and lipolysis (88, 95). Different from other type 2 cytokines, ablation of IL-10 elicits thermogenesis and browning of scWAT and protects against diet-induced obesity. ATAC-seq, ChIP-seq, and RNA-seq analyses revealed that IL-10 affects chromatin structure and CCAAT/enhancer binding protein-β (C/EBPβ) and activating transcription factor 2 (ATF2) occupancy at the promoters of thermogenic genes (96).

### M1 macrophages in adaptive thermogenesis and lipolysis

In contrast to M2 macrophage, M1 macrophage and its secreted proinflammatory cytokines usually exert negative effects on thermogenesis and lipolysis. Prolonged treatment with TNF cytokine inhibits the sensitivity of adipocytes to β-adrenergic stimulation, and thus inhibits *Ucp1* gene expression, thermogenesis and lipolysis (97, 98). TNF-activated inhibitor of nuclear factor kappa-B kinase subunit epsilon (IKKϵ) and TANK-binding kinase 1 (TBK1) desensitize lipolytic signaling by phosphorylating and activating phosphodiesterase 3B (PDE3B), which decreases cAMP levels (98, 99). Overexpression of IKKϵ blunts β-adrenoreceptor-stimulated *Ucp1* expression in adipocytes, while an inhibitor of IKKϵ and TBK1 restores catecholamine sensitivity and reversed the effects of HFD feeding on thermogenesis and weight gain (98, 100). IL-1β inhibits β-adrenoreceptor-stimulated *Ucp1* expression which was significantly abrogated by the inhibition of ERK (101, 102). Genetic ablation of *Jnk1*, a major intracellular mediator of inflammatory signaling, enhances *Ucp1* expression and thermogenesis in adipose tissues (103). Through singly or in combination treatments of beige adipocytes *in vitro* with different proinflammatory cytokines, we found that TNF and IL-1β moderately inhibited adrenergic signaling separately, while a mixture of four cytokines (IL-1α, IL-1β, IL-6 and TNF) achieved a dramatic inhibition of thermogenesis and lipolysis (47). Besides proinflammatory cytokines, adipocytes express TLRs and key components of their downstream signaling pathway. LPS or palmitic acid-stimulated TLR4 signaling abolished cAMP-induced upregulation of *Ucp1* and thermogenesis through activating NF-κB and MAPK pathways (104, 105). Consistently, TLR3 and TLR4, upstream of interferon regulatory factor 3 (IRF3) signalling, induce insulin resistance and thermogenesis in adipocytes (106). IRF3-deficient mice exhibit systemic inflammation and enhanced browning of scWAT when fed HFD (107). Inflammation-imposed inhibition of beige adipogenesis and thermogenesis are also mediated by a direct adhesion of inflammatory macrophages to adipocytes. Specifically, α4 integrin-mediated adhesion of inflammatory M1 macrophages to VCAM-1, which is expressed by adipocytes, inhibits thermogenesis in an ERK-dependent manner. Genetic or pharmacologic inhibition of α4 integrin resulted in an increase of beige adipogenesis and UCP1 expression of the scWAT (93).

ATM also regulates adipocyte thermogenesis and lipolysis indirectly through other cells like sympathetic nerves. Fasting and cold exposure increase the release of catecholamines from sympathetic nerves, which bind to adipocyte adrenergic receptors and activate cAMP-PKA signaling to trigger lipolysis and thermogenesis. Yochai Wolf et al. reported a homeostatic role of macrophages in the control of brown adipose tissue innervation in mice (108). They found that BAT resident CX3CR1^+^ macrophages inhibit sympathetic innervation and decrease the local level of catecholamine. *Mecp2* deficiency in CX3CR1^+^ macrophages decreased thermogenesis and led to spontaneous obesity (108). Two recent reports identified a subtype of macrophages in human and mouse WAT that take up and degrade norepinephrine (NE), then inhibit adipocyte lipolysis and thermogenesis. These macrophages are termed either sympathetic neuron associated macrophages (SAMs) or nerve-associated macrophages (NAMs). Different from CX3CR1^+^ macrophage that inhibits sympathetic neuronal innervation in BAT, SAMs/NAMs function in eWAT and scWAT (109, 110). Mechanistically, NE-degrading macrophages are activated *via* NLR family pyrin domain containing 3 (NLRP3) inflammasome system in aged WAT. NLRP3 activation upregulates the expression of growth differentiation factor-3 (GDF3) and GDF3-dependent expression of monoamine oxidase A (MAOA) that degrade NE. Macrophages that lack NRLP3 or GDF3 decreased adipose NE removal and increased lipolysis upon aging. The MAOA inhibitor treatment of aged mice restored fasting-induced lipolysis and increased expression of UCP1 (110). Moreover, activin receptor-like kinase 7 (ALK7) signaling, which is activated by GDF3, contributes to diet-induced catecholamine resistance in adipose tissue. Fat-specific *Alk7* knock-out enhances adipose β-adrenergic signaling, lipolysis and thermogenesis, resulting in reduced fat mass and resistance to HFD-induced obesity (111). SAMs in mouse WAT can specifically express the NE transporter SLC6A2. Genetic ablation of *Slc6a2* in SAMs increases thermogenesis and weight loss in obese mice (109). CX3CR1^+^ macrophages produce IL-27 to activate p38 MAPK-PGC-1α pathway in adipocytes and promote thermogenesis of BAT and scWAT (112). However, it is unknown whether these SAMs/NAMs can be categorized as M1 or M2.

## ATM in insulin resistance and diabetes

Type 2 diabetes is associated with obesity and occurs as a consequence of insulin resistance, which emerges when three major insulin-sensitive tissues (skeletal muscle, liver, and adipose tissue) can not respond well to insulin and can not effectively take up glucose from blood. Insulin is a peptide hormone secreted by pancreatic β cells, which plays a crucial role in carbohydrate metabolism, lipid anabolic regulation, cell growth and proliferation (113). Blood glucose induces β-cells to produce and secrete insulin, which stimulates glucose uptake in different types of cells, including adipocyte, muscle cell, liver cell and others, thereby decreasing blood glucose level. The effects of insulin on whole-body metabolism result from its binding to insulin receptor (IR), leading to autophosphorylation of specific tyrosine residues of IR and subsequently phosphorylation of proteins known as insulin receptor substrates (IRS). PI3K, a key component of IRS downstream, mediates insulin signaling mainly by activating PKB/AKT and PKC signaling pathways (114).

### TNF

Adipose tissue macrophage modulates insulin action through different mechanisms, with M1 macrophage promoting insulin resistance while M2 macrophage enhancing insulin sensitivity (115, 116). The major difference between M1 and M2 is the expression of proinflammatory cytokines. In 1990s, the first study reported the inflammatory origin of obesity and diabetes. They found that adipose tissue from different obese rodents and humans has an enhanced secretion of proinflammatory cytokines, mainly TNF, which was linked to insulin resistance (117, 118). TNF‐deficient obese mice are protected from obesity‐induced insulin resistance in muscle and adipose tissues (119). The important role of TNF is further evidenced by TNF neutralization, which improves an increased peripheral glucose uptake and insulin sensitivity in obese mice (117). These studies showed that blocking a single cytokine can restore insulin sensitivity, and macrophage was further identified as the major cell source of TNF and other proinflammatory molecules in obesity (4). Binding of TNF to its receptors results in the activation of JNK and causes phosphorylation of IRS1 at serine 307, which impairs IR-mediated tyrosine phosphorylation of IRS1 and downstream signaling (120).

### IL-1β

IL-1β is another important proinflammatory cytokine that is produced by ATM (121). IL-1β exerts its biological effects by binding directly to IL-1Rα and activates the IKK/NF-κB pathway (122). It was reported that adipose tissue appears to be a major source of IL-1R antagonist production, which prompted an interest in the role of IL-1β in obesity-induced diabetes and insulin resistance (123). IL-1β, released by ATM, alters insulin sensitivity of adipose tissue by inhibiting insulin signaling, so it decreases insulin-stimulated glucose uptake and lipogenesis in both murine and human adipocytes (124, 125). *In vitro* studies revealed that IL-1β treatment of adipocytes disturbs insulin signaling *via* downregulation of IRS1 expression, leading to a reduction of translocation of insulin-stimulated glucose transporter type 4 (GLUT-4), an essential process for glucose uptake (124, 125). Consistently, insulin resistance of human adipocytes imposed by macrophage-derived conditioned medium can be reversed by neutralizing IL-1β (124).

### IL-6

Many studies have established a positive correlation between IL‐6 and insulin resistance (126). Adipose tissue‐derived IL‐6 enters circulation and exerts systemic regulation on insulin action. Up to 35% of systemic IL‐6 originates from adipose tissue under basal condition, secreted by both adipocytes and macrophages (4, 127). IL-6 has been described to impair insulin signaling, primarily through inhibiting insulin-stimulated tyrosine phosphorylation of IRS in adipose tissue (128). Modest increase of basal glucose transporter GLUT1 was observed in 3T3‐L1 adipocytes when incubated with IL‐6 (129), while the expression of *Glut4* and *Irs1* genes was inhibited by chronic IL‐6 treatment (128, 130). Besides, IL‐6 induces the expression of SOCS3, which is a negative regulator of insulin signaling in adipocytes (131).

### NF-κB

ATM-released proinflammatory cytokines activate different signaling pathways in adipocytes to modulate insulin action. NF-κB is a master inflammatory transcriptional factor involved in a variety of physiological and pathological processes such as inflammation and innate and adaptive immune responses. The activation of NF-κB signaling can increase the expression of several proinflammatory genes, which exacerbate insulin resistance progression (132, 133). IKKβ specific deficiency in adipocytes completely prevents FFA-induced IL-6 and TNF expression, and improves glucose tolerance and insulin sensitivity (134, 135). Mechanistically, IKKβ activation promotes IRS1 serine phosphorylation through activation of the TSC1/TSC2/mTORC1/S6 kinase-1 pathway, which impairs IR-mediated tyrosine phosphorylation of IRS1 (136). In addition, activation of the IKKβ/NF-κB pathway increases the expression of protein-tyrosine phosphatase 1B (PTP1B), a tyrosine phosphatase that catalyzes dephosphorylation of tyrosine residues of IRS1, further inhibiting insulin signaling in adipose tissue (137).

### JNK

JNK might be the most investigated stress kinase in obesity-related insulin resistance. The activity of JNK in increased upon exposure to inflammatory stimuli which include cytokines, FFAs, and then phosphorylates transcription factor activator protein-1 (AP-1) (120). Like IKKβ, JNK inhibits insulin signaling through an inhibitory serine-threonine phosphorylation of IRS1, thereby decreases PI3K/AKT signaling (138, 139). It should be noticed that JNK seems more implicated in the direct regulation of IRS serine phosphorylation than IKKβ (140). JNK activity could be induced in adipose tissue of obese mice compared to lean mice. Adipose tissue-specific JNK1-deficient mice are protected against the development of insulin resistance under HFD feeding. Interestingly, this protective effect is not systemic as JNK1 deficiency in adipocytes only restore liver but not muscle insulin sensitivity (141). Besides JNK1, JNK2 isoform is also involved in insulin resistance but to a lesser extent (142). Moreover, using a myeloid cell-specific JNK1/2 double knock-out mouse model, another study demonstrated that macrophage JNK1/2 are required for the establishment of obesity-induced adipose tisse inflammation and insulin resistance through promoting macrophage M1 activation (143).

### ERK1/2

**Figure 3:**
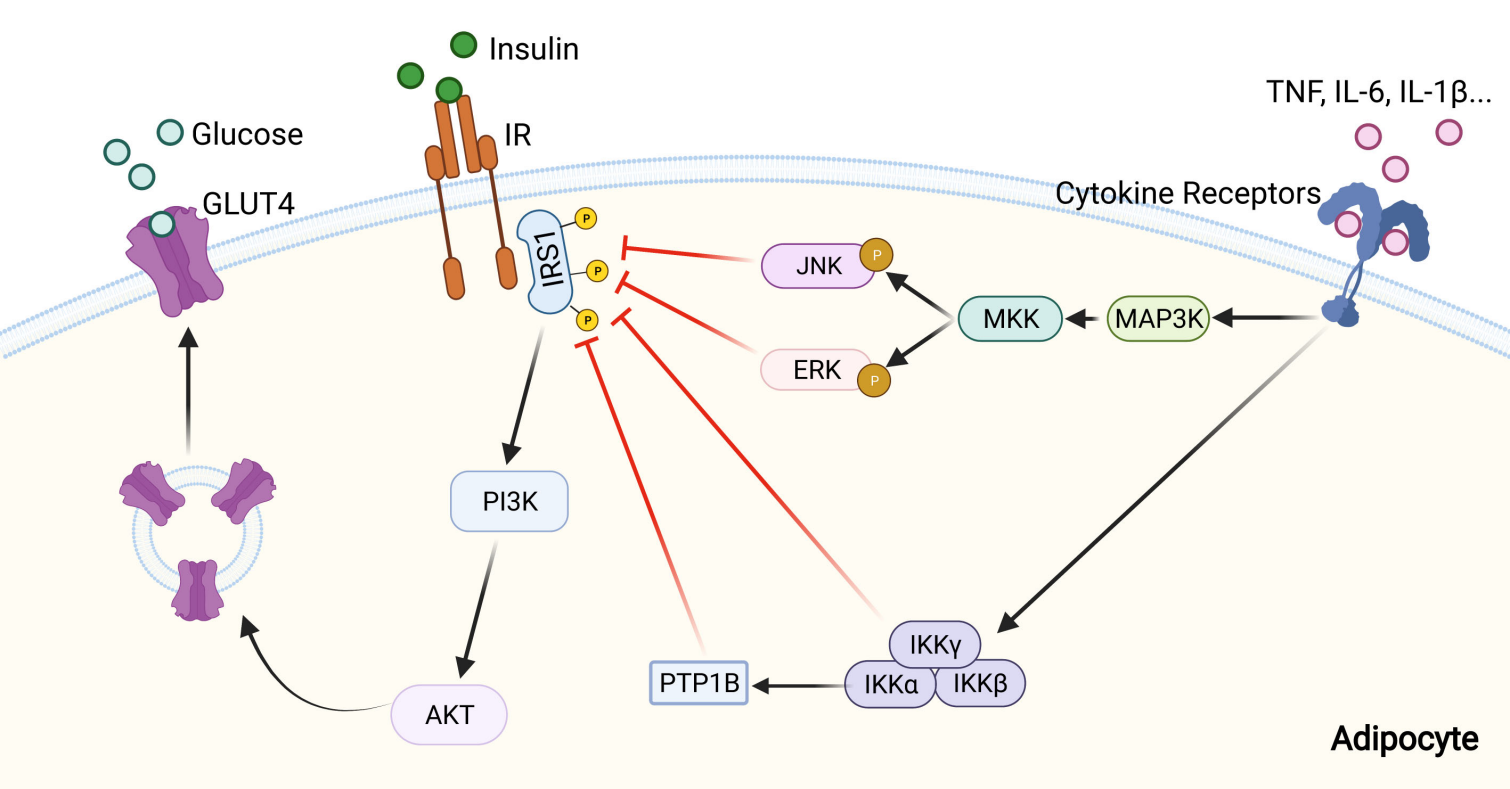
Inhibition of insulin signaling pathway by inflammatory signaling. Pro-inflammatory cytokines, including IL-1β, IL-6, and TNF, can activate inflammatory signaling pathways through their receptors. Then activated MAPK and NF-κB signaling pathways inhibit insulin signaling by altering the phosphorylation status of IRS-1, and further lead to a reduction of glucose uptake by adipocyte.

ERK1/2 is activated in adipose tissue of obese mice or human (144). Multiple cellular studies have reported that activated ERK1/2 in diabetes induces IRS1 serine phosphorylation, which inhibits IRS1 tyrosine phosphorylation. In addition this serine phosphorylation decreases the interaction between IRS1 and PI3K and inhibits the association between IRS1 and insulin receptor, further diminishing the metabolic effects of insulin (140). ERK1-deficient mice are protected against diet-induced obesity and insulin resistance by inhibiting adipogenesis and promoting energy expenditure (145). Besides, ERK activation promotes insulin resistance indirectly, mainlythrough a stimulation of adipocyte lipolysis and FFA release, and mice deficient in the signaling adapter p62 (an ERK inhibitor) show similar phenotypes (**Figure 3**) (146, 147).

The regulation of insulin sensitivity by ATMs is also mediated by miRNAs, which are contained and transferred by ATM-derived exosome (148). Treatment with obese ATM-derived exosome leads to insulin resistance, whereas lean ATM-derived exosome increases insulin sensitivity in obese mice. Mechanistically, miR-155 is among the differentially expressed miRNAs in obese ATM-derived exosome, and it causes systemic insulin resistance and glucose intolerance (148).

In addition to M1 and M2 macrophages, other macrophage populations that regulate insulin resistance have been reported recently, including neuropilin-1 (NRP1)^+^ macrophages and TREM2^+^ lipid-associated macrophage (LAM) (149, 150). NRP1^+^ macrophages accumulate in adipose tissue and protect against obesity and metabolic syndrome. Conditional deletion of NRP1 in macrophages compromised lipid uptake and led to insulin resistance (149). Using single-cell RNA sequencing, one study identified a subset of TREM2^+^ lipid-associated macrophages (LAMs) that prominently arise under obesity condition both in humans and mice. TREM2 deficient mice showed inhibited recruitment of macrophages to CLS and led to adipocyte hypertrophy as well as insulin resistance (150). However, bone marrow transplantation experiments by another group argued that hematopoietic-expressed TREM2 is dispensable for obesity-induced metabolic dysfunction, including insulin resistance (151).

## ATM in fibrosis

Adipocytes are surrounded by a network of extracellular matrix (ECM) proteins that not only serve as a structural support and protection but also regulate adipose tissue homeostasis by responding to different signals (152). In other words, ECM ensures adipose tissue to expand and maintain in a healthy manner. However, the development of obesity promotes an excessive accumulation of ECM proteins and elicits adipose tissue fibrosis, which reduces tissue plasticity and results in adipocyte dysfunction such as insulin resistance (153, 154). Thus, fibrosis is considered as a hallmark of metabolically dysfunctional adipose tissue.

During the development of obesity, rapid adipose tissue expansion and adipocyte enlargement cause adipose tissue hypoxia and lead to an activation of hypoxia-inducible factor 1-alpha (HIF1-α), which induces the production of ECM proteins (155, 156). Hypoxia promotes expression of many proinflammatory genes through HIF1-α induction. Low-grade inflammation contributed by hypoxia in obesity further deteriorates adipose tissue fibrosis (157).

In addition, adipocyte hypertrophy and adipose tissue hypoxia are tightly associated with increased infiltration of macrophages, which promote local ECM accumulation (154, 158). Proinflammatory M1 macrophages make up significant proportion in fibrotic adipose tissue. Besides proinflammatory cytokines, ATM produces many other cytokines, including TGF-β1 and PDGF, which directly activate fibroblasts and increase ECM accumulation (159). As a vicious spiral, macrophages promote fibrogenesis by releasing chemokines that attract fibroblasts and more proinflammatory cells (160). Besides, macrophage inducible C-type (Mincle), which is induced in macrophage by TLR4 activation, regulate ECM prodaction and degredation, as well as fibroblast proliferation (161, 162). Additionally, saturated fatty acids (SFA)-mediated inflammation is potentiated by TLR4 activation and contributes to WAT fibrosis by fueling local inflammation (31). Besides their role in promoting fibrogenesis, macrophages participate in ECM clearance through collagen uptake and degradation. Collagen phagocytosis by macrophages depends on mannose receptor 1 (MRC1) and urokinase plasminogen activator receptor-associated protein (uPARAP/endo180) (163). In adipose tissue, it has been widely accepted that there is no single signaling or single cell type responsible for ECM production. Adipose tissue collagens are contributed by both preadipocytes and macrophages, and the fibrosis is coordinated through intimate crosstalk between macrophages and preadipocytes under different physiological and pathological conditions (164). *In vitro* studies suggest that preadipocytes in contact with proinflammatory macrophages can produce ECM proteins, including collagen I and fibronectin (164). Meanwhile, macrophage is found to be the master regulator of fibrosis through producing TGF-β1 and PDGF, which have been proved to directly activate fibroblasts and control ECM dynamics by regulating the balance between various matrix metalloproteinases (MMPs) and their inhibitors (159).

## ATM and adipokines

Adipose tissue secretes many kinds of hormones, called adipokines, which exert their biological functions in an autocrine, paracrine, and/or systemic manner and influence many physiological/pathological processes, such as thermogenesis, insulin resistance and fibrosis (165). The most well-known adipokines are adiponectin and leptin, both of which can exhibit either proinflammatory or anti-inflammatory property, thereby contributing to adipose tissue functions (165).

### Adiponectin

Adiponectin, a 30-kDa adipokine exclusively secreted from adipocytes, exists in cells and plasma (166, 167). As the most aboundent peptide secreted by adipocytes, adiponectin shows protective activity in multiple diseases such as inflammation, obesity and insulin resistance (166, 168–170). Many evidences proved that adiponectin acts as anti-inflammatory factor by regulating the polarization of adipose tissue macrophages (36, 104). Recombinant adiponectin treatment results in an increased expression of M2 markers and a decreased expression of M1 markers in adipose tissue, while macrophages from adiponectin knock-out mice display increased M1 markers (36, 171). Interestingly, adiponectin has also been reported as a proinflammatory factor to increase TNF-α and IL-6 secretion directly. The authors further suggested that the anti-inflammatory property of adiponectin may be due to its desensitized effects on cells for further proinflammatory response, although the specific molecular mechanism is still unknown (172, 173). Additionally, adiponectin has been reported to induce adipose tissue M2 macrophage proliferation both *in vivo* and *in vitro*, further promoting cold-induced adipose tissue thermogenesis (174). Mechanistically, adiponectin is recruited to the cell surface of M2 macrophages *via* T-cadherin and promotes cell proliferation by activation of AKT signaling (174). Besides, several intracellular signaling pathways have been reported to mediate adiponectin action in regulating macrophages. Adiponectin suppresses M1 macrophage proliferation *via* inhibiting NF-κB signaling (175). A mutual antagonistic action was observed between adiponection and TNF/IL-6 expression. LPS-induced TNF/IL-6 is suppressed by adiponectin, and TNF/IL-6 conversely inhibit adiponectin expression (176, 177). Besides, both oxidative stress and ROS release inhibit adiponectin expression in obesity, therefore forming a vicious circle that lowers adiponectin level while increases proinflammatory cytokines and oxidative stress in obese adipose tissue (56).

### Leptin

Leptin, another pivotal adipokine, exerts its function through modulating immunity and inflammation (178). Leptin is a 16-kDa peptide hormone secreted mainly from adipose tissue, and the most evident function of leptin is its control of energy balance by inhibiting appetite through hypothalamus (179, 180). However, leptin receptor (LEPR) is ubiquitously expressed on the surface of many cells like immune cells, suggesting pleiotropic actions of leptin (181, 182). High levels of thymocyte apoptosis and reduced thymic cellularity were observed in in obese mice with mutation in leptin (*ob/ob* mice) or LEPR (*db/db mice*), which were reversed by peripheral administration of recombinant leptin, revealing an important role of lepin in immunity (183). Consistently, *ob/ob* mice show impaired cellular and humoral immue activities, and they are protected against inflammation in different models (184–186). Besides acting on adaptive immunity, leptin regulates innate immune cells such as macrophages, to promote inflammation. Macrophages generated from *ob/ob* or *db/db* mice showed a decrease of phagocytosis and inflammatory cytokine production, whereas exogenous leptin administration upregulated both of them (178, 181). Several *in vitro* or *ex vivo* studies in wild-type mice also support that leptin acts as a proinflammatory factor in immune cells. They showed that exogenous leptin administration upregulated both phagocytosis and production of proinflammatory cytokines (4, 187–189). Leptin stimulates production of proinflammatory cytokines through activation of JAK2-STAT3 pathway in macropahges (189, 190). Moreover, leptin-deficient mast cells polarize macrophages from M1 to M2 and thus protects mice from obesity (191). Leptin has proinflammatory properties, and the expression of leptin in adipose tissue as well as circulating leptin are promoted by administration of proinflammatory stimuli (192, 193). Thus, it appears that proinflammatory cytokines and leptin form a vicious circle that promotes the development of chronic inflammation and obesity.

## Conclusions and perspective

**Figure 4:**
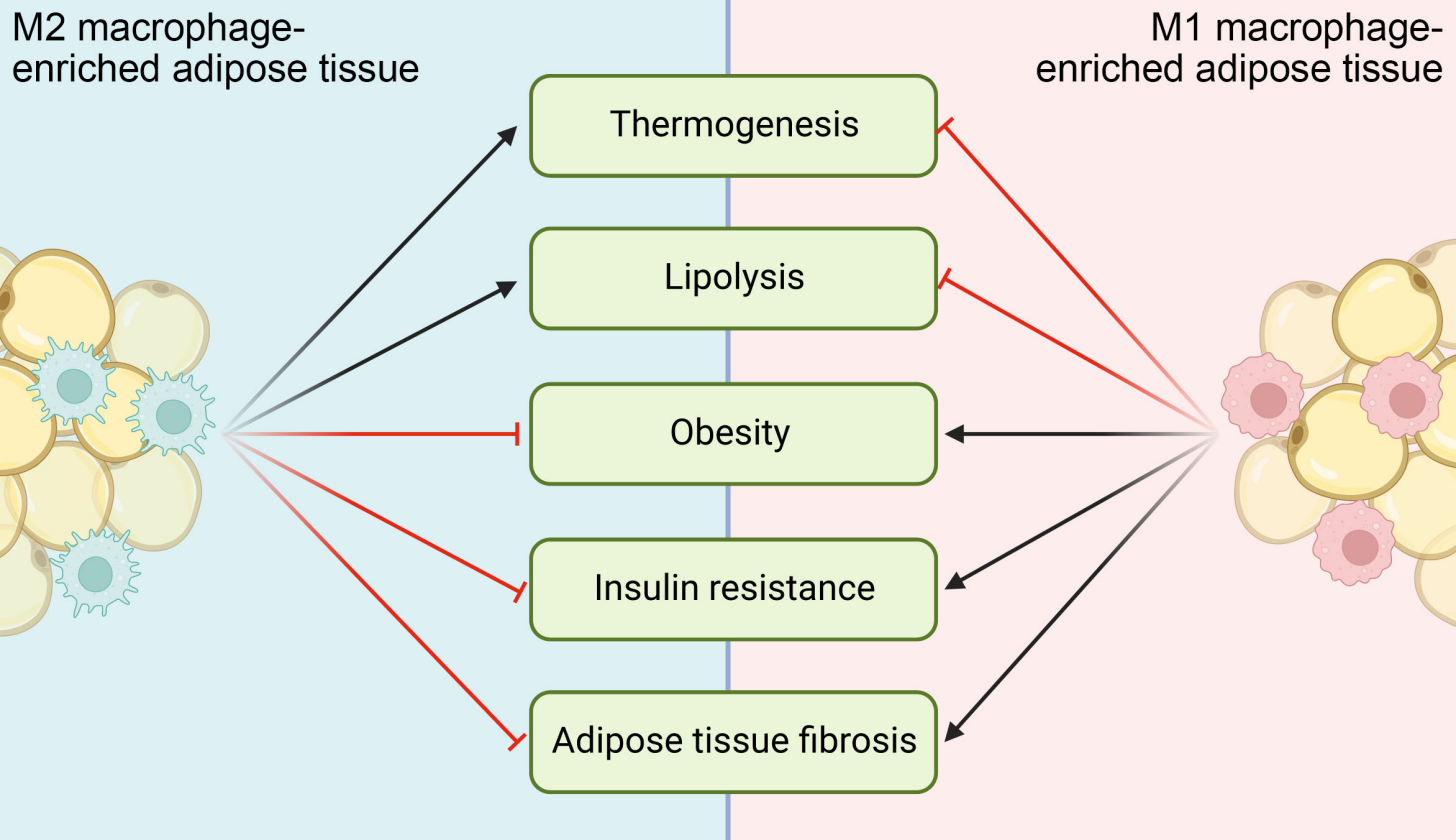
Functional implications of ATMs in different metabolic diseases. M1 ATMs inhibit adipocyte lipolysis and thermogenesis, while M2 ATMs do the opposites. When M1 activation of ATMs chronically exceeds M2 activation, metabolic diseases like obesity, insulin resistance and adipose tissue fibrosis ensue and/or deteriorate.

Macrophage is the most abundant cell population and believed to play a dominant role in the homeostasis of adipose tissue and whole-body energy metabolism, whose dysregulation significantly contributes to metabolic diseases (**Figure 4**). Noticebaly, many other immune cells exist in adipose tissue as well, including both innate and adaptive immune cells like ILC2s, eosinophils, invariant natural killer T (iNKT) and T lymphocytes. They play critically important roles in the maintenance of energy homeostasis and contribute to the metabolic dysfunction, directly or indirectly through crosstalk with macrophages (194). ILC2s recruit and activate eosinophils through IL-5, and then eosinophil-secreted IL-4 and IL-13 promote macrophage M2 activation. Besides, ILC2s directly secret IL-13, which induces a physiological expansion and differentiation of beige adipocyte precursors, further promoting adipose tissue thermogenesis (95). iNKT cells are a type of innate immune cell, and their activation induces a production of fibroblast growth factor 21 (FGF21) that promotes thermogenic browning of WAT (195). Infiltration of CD8^+^ T lymphocytes is an early event during the development of obesity and contribute to macrophage accumulation. Adoptive transfer of CD8^+^ T cells into CD8-deficient obese mice induces M1 macrophage infiltration and promotes systemic insulin resistance (196).

One goal of the mouse ATM investigation is to exploit its specific characteristics and functions to treat human metabolic diseases. Even though ATMs from human and mouse models are highly similar in both gene expression pattern and function, considerable differences exist (197, 198). Mouse models have their own limitations and can not replicate the properties of humans in many aspects, thus the validation of findings from mouse models in humans is critical for their translation. But, there are still many challenges for mouse-to-human validation because of the technical limitations, including limited tools to safely manipulate ATM in humans.

The great progress of ATM studies generates many further questions that need to be addressed in the future. Firstly, it is difficult to manipulate ATM specifically. Several genetically modified animal models are used to investigate tissue-specific macrophages, including *Lyz2*-Cre, *Cx3cr1*-Cre, *CD11b*-Cre and *F4/80*-Cre, whereas all of them are ubiquitously expressed in macrophages from different tissues but not specifically in ATM (199). Besides, some of them are expressed in other types of cells. For example, the widely used *Lyz2*-Cre is expressed in other myelomonocytic cells, including most granulocytes, few CD11c^+^ dendritic cells (DCs), and a small percentage of non-hematopoietic cells (200). Thus, more specific markers and animal models need to be identified and established, which will greatly facilitate our understanding on ATM in health and metabolic diseases. Secondly, macrophage constitutes a plastic and heterogeneous cell populations modulated by and interacted with their microenvironment in different adipose tissues. Distinct fat pads in different locations show different molecular, cellular and anatomical features (201). Accordingly, the physiological characteristics of ATM in these adipose tissues may be quite diverse, which have not been well investigated yet. Thirdly, ATM was oversimplified to be divided into two groups, M1 and M2 macrophages. However, this M1/M2 classification has been questioned as a result of the identifications of many distinct ATM subtypes, including CD9^+^ macrophage (202), TREM2^+^ LAM (150) and SAM (109). Although single cell RNA-sequencing provides an objective view on the identity and function of ATM (150), more unbiased approaches and new technologies should be used to identify and characterize all the different ATM populations and their regulations on health and metabolic diseases.

## Author contributions

JY wrote the manuscript and DW and YQ edited it. All authors approved the submitted version. All authors contributed to the article and approved the submitted version.

## Funding

This work was supported by grants from National Key R&D Program of China (2018YFA0800702 and 2021YFA0804801), National Natural Science Foundation of China (31671227 and 91642113) and the Thousand Young Talents Program of the Chinese government (to YQ).

## Acknowledgments

Figures were created using BioRender.com.

## Conflict of interest

The authors declare that the research was conducted in the absence of any commercial or financial relationships that could be construed as a potential conflict of interest.

## Publisher's note

All claims expressed in this article are solely those of the authors and do not necessarily represent those of their affiliated organizations, or those of the publisher, the editors and the reviewers. Any product that may be evaluated in this article, or claim that may be made by its manufacturer, is not guaranteed or endorsed by the publisher.
